# Recalcitrant Issues and New Frontiers in Nano-Pharmacology

**DOI:** 10.3389/fphar.2019.01369

**Published:** 2019-11-29

**Authors:** Vinay Bhardwaj, Ajeet Kaushik, Ziad M. Khatib, Madhavan Nair, Anthony J. McGoron

**Affiliations:** ^1^Department of Biomedical Engineering, The College of New Jersey, Ewing, NJ, United States; ^2^Department of Natural Sciences, Florida Polytechnic University, Lakeland, FL, United States; ^3^Division of Hematology Oncology, Department of Pediatrics, Nicklaus Children’s Hospital, Miami, FL, United States; ^4^Center for Personalized Nanomedicine, Herbert Wertheim College of Medicine, Florida International University, Miami, FL, United States; ^5^Department of Biomedical Engineering, Florida International University, Miami, FL, United States

**Keywords:** nanoparticles, theranostics, research and development, nano-based drugs, Doxil, nanomedicine

## Abstract

Packaging of old pharma drugs into new packaging “nanoparticles” is called nano-pharmacology and the products are called nano-based drugs. The inception of nano-pharmacology research and development (R&D) is marked by the approval of the first nano-based drug Doxil^®^ in 1995 by the Food and Drug Administration. However, even after more than two decades, today, there are only ∼20 nano-based drugs in the market to treat cancers and brain diseases. In this article we share the perspectives of nanotechnology scientists, engineers, and clinicians on the roadblocks in nano-pharmacology R&D. Also, we share our opinion on new frontiers in the field of nano-pharmacology R&D that may allow rapid and efficient transfer of nano-pharma technologies from R&D to market.

## Introduction

The National Cancer Institute Surveillance, Epidemiology, and End Results Program has reported a significant decrease in cancer deaths in the USA (Ward et al., 2019). However, cancer is still the second leading cause of deaths worldwide, with low and middle income countries constituting 70% of global cancer deaths (World Health Organization, 2018). In contrast, 11% of the total global burden of neurological disorders is found in high income countries that typically have a more aging population (World Health Organization, 2011). Among innovative healthcare technologies to diagnose and treat cancer and brain diseases, nano-pharmacology has found a special place. Nano-pharmacology involves the application of nanoparticles to improve the pharmacokinetics or efficacy of drugs to 1) reach their target site, 2) minimize their side effects, 3) improve their bioavailability (dissolution and solubility rate), and other benefits. Nano-pharmacology in simple words involves packaging drugs, such as chemo- and immuno-therapeutic agents, nucleic acids, and small molecules inside nanoparticles.

As of today, there are around 20 commercial nano-based drugs for oncology and neurology that are either approved by the Food and Drug Administration (FDA) or likely to be approved by the FDA as they have completed clinical phase III trials (**Table 1**). More than 80% of these FDA-approved nano-based drugs are based on liposomes and polymers and intended for cancer therapy. However, this number is not significant if we compare the return with respect to time and money invested in the last three decades since the development of the first nano-based drug Doxil^®^ (Barenholz, 2012). On evaluating the properties of commercial nano-based drugs (**Table 1**) (Etheridge et al., 2013; Hua et al., 2018), we find that the core of the technology and obstacles in their progress has remained the same as it was during the translation of Doxil^®^. The technology behind most of the commercial products and those under clinical trials is based on using liposomes or polymers to encapsulate drugs inside their core and utilize intravenous injection to let the nano-formulation diffuse passively to the disease site primarily based on a combination of their size, shape, and charge (Barenholz, 2012; Hua et al., 2018).

**Table 1:** List of commercial (FDA-approved) and likely-to-be commercial (completed phase III clinical trial) nano-based drugs in USA to treat cancer and brain diseases. Adapted from Reference 5 and 6.

Name	Company	Material	Drug	Disease/s	Approved
Doxil/Caelyx	Janssen	Liposome	Doxorubicin	Kaposi sarcoma Ovarian cáncer Multiple myeloma	1995 2005 2008
DaunoXome	Galen Pharma	Liposome	Daunorubicin	Kaposi sarcoma	1996
DepoCyt	Sigma Tau	Liposome	Cytarabine	Lymphomatous meningitis	1999
Myocet	Elan Pharma	Liposome	Doxorubicin	Breast cancer	2000
Lipodox (generic Doxil)		Liposome	Doxorubicin	Same as Doxil	2013
Marqibo	Onco TCS	Liposome	Vincristine	Acute Lymphoma Leukemia	2012
Onivyde	Merrimack	Liposome	Irinotecan	Pancreatic cancer	2015
Vyxeos	Jazz Pharma	Liposome	Daunorubin Cytarabine	Acute Myeloid leukemia	2017
Oncaspar	Enzon Pharma	Polymer	.Aspargase	Acute Lymphoblatic eukemia	1994
Copaxone	Teva	Polymer	Glatiramer acetate (synthetic protein)	Multiple sclerosis	1996
Eligard	Tolmar	Polymer	Leuprolide acetate	Prostate cancer	2002
Plegridy	Biogen	Polymer	PEG-Interferon beta-1a	Multiple sclerosis	2014
Glatopa (Generic Copaxone)	Novartis	Polymer	Glatiramer acetate (synthetic protein)	Multiple sclerosis	2015
Ontak	Cisai Inc	protein	Denileukin diftitox (Synthetic protein)	Cutaneous T-cell lymphoma	1999
Abraxane	Celgene	protein	Paclitaxel	Breast Cancer NSCLC Pancreatic cancer	2005 2012 2013
Invega Sustenna	Janssen	nanocrystals	Paliperidone Palmitate	Schizophrenia	2009
Nanotherm	MagForce	Inorganic	Iron oxide	Glioblastoma	2010
Onpattro	Alnylam Pharmaceuticals	Lipid/Liposome	Patisiran (ALN-TTR02)	hATTR Amyloidosis	2018
ThermoDox	Celsion	Liposome,	Doxorubicin	Hepatocellular carcinoma	Phase III completed ClinicalTrials.gov identifier#NCT00617981
Paclical	Oasmia Pharma	Polymer	Paclitaxel, Doxil	Ovarian cancer	Phase III completed. ClinicalTrials.gov identifier# NCT00989131
NK-105	Nippon Kayaku	Polymer	Paclitaxel	Breast cancer	Phase III completed ClinicalTrials.gov identifier# NCT01644890

We will share our opinion on some major recalcitrant issues behind the slow progress of nano-based drugs from R&D to market, and share some thoughts on new frontiers in nano-pharmacology research with some examples to persuade scientists and engineers to explore new routes in nano-pharmacology research. The obstacles listed below can be categorized as technical (poor reproducibility, and poor targeting and efficacy), fiscal (revenue with respect to investment), and regulatory issues.

### Recalcitrant Issues and New Frontiers

#### Poor Reproducibility in Humans

Animal testing is critical to investigate any medicine, including nanomedicines, before they can be tested in humans. However, in our opinion, insufficient attention has been paid to differences between animal and human models of disease and their response to potential therapies. Two key points cannot be emphasized enough. First, mice are mice and humans are humans, and many aspects of human disease cannot be modeled in animals. Second, there is no doubt that successful treatment of a disease in rat and mouse models using a nano-formulation appears encouraging, however, there are many flaws and potential human harm associated with relying too much on safety studies done on animals (Akhtar, 2015). The former Director of the NIH, Dr. Elias Zerhouni has commented “We have moved away from studying human disease in human…, we all drank the Kool-Aid! The researchers have over-relied on animal data.” “The problem is that it hasn’t worked, and it’s time we stopped dancing around the problem…. We need to refocus and adapt new methodologies for use in humans to understand disease biology in humans” (McManus, 2013). There is a global effort to establish the Alternatives to Animal testing program with a goal to develop and validate alternative analytical methods to Replace, Reduce and Refine (3 Rs) use of animals in research (Burgdorf et al., 2019).

#### Need for Improved Analytical Tools

Another recalcitrant issue in nano-pharmacology research in the pre-clinical and clinical phases is the analytical methods used for safety and efficacy monitoring. More than half of the drugs in phase III clinical trials fail, and the primary reasons are inadequate efficacy (57% of the failed drugs) and safety concerns (17% of the failed drugs) (Hwang et al., 2016). For decades, we have been practicing classical analytical technologies to validate safety (targeted delivery of nano-formulation to disease site) and efficacy (drug release from nano-formulation to the disease site). These analytical technologies are either destructive and *ex-vivo* (chromatography, mass spectroscopy, histopathology, etc.) or indirect (fluorescence to measure fluorophore conjugated to drug). Destructive and indirect methods of measurements can lead to uncertainty in measurement of the drug which could be one of the many reasons why many medicines fail in clinical trials. There is no quantitative analytical method available in the clinic to in real-time measure the bio-distribution of nanoparticles, except with radio-labeling, a label-based imaging technology that could lead to unreliable results and has safety concerns of its own (Sanhai et al., 2008). There is a need for analytical technologies that can in real-time, non-invasively and label-free, monitor the complete process, from tracking of the nanoparticles in the body, to drug release at the disease site, and finally the clearance of the nanoparticles from the body without side-effects. We do not want to overstretch by proposing development of a single technology that can monitor the complete process. However, there is definitely a need for more research in the development of alternative more effective analytical technologies in this direction.

Currently, there are six clinical standard analytical technologies to image cancer: X-ray with and without computed tomography (CT), ultrasound, magnetic resonance imaging (MRI), positron emission tomography (PET), single-photon emission computed tomography (SPECT), and optical imaging (Frangioni, 2008). Among the six, optical imaging is fast, safe, inexpensive, ultra-sensitive, specific, and highly quantitative (Frangioni, 2008), making it one of the most favorable technologies for rapid screening and therapy response monitoring. To achieve the goal of eradicating cancer from society, we need to develop ultra-sensitive and targeted analytical technologies that can detect single malignant cells, and monitor drug release at the cellular level. Compared to the aforementioned technologies, optical imaging, particularly near-infrared (NIR) imaging (generally accepted to be between 650 and 950 nm), is most sensitive and quantitative, and can be targeted to single cells and even intracellularly by using exogenous labels like fluorophores to achieve molecular imaging of the drug release and the tumor microenvironment (Frangioni, 2008). Let us take two successful examples of optical spectroscopy or imaging technologies tested on cancer and brain disease patients. Breast cancer, which is the most common type of cancer in women worldwide, was screened with 85% accuracy in ∼2,000 women using NIR imaging (Leff et al., 2008). NIR optical imaging and ultrasound are the most rapid, safe, affordable, and portable among all existing and emerging technologies to screen breast cancer (Godavarty et al., 2015). X-ray mammography and ultrasound give structural information, while optical imaging has the advantage of giving functional information (Godavarty et al., 2015). The functional information on tumor microenvironment at the molecular level can be very useful in monitoring therapy response. Raman spectroscopy is a non-invasive, portable, simple, and rapid technology that can fit in the palm of a neurosurgeon’s hand to guide them during surgery to accurately resect a tumor in human patients (Jermyn et al., 2015; Brusatori et al., 2017; Desroches et al., 2018; Zhang et al., 2019), and at the same time it has the potential to track nanoparticles and drug release without the requirement to conjugate a label to the drug or the nanoparticle (Kang et al., 2013; Yang et al., 2014; Srinivasan et al., 2016).

A critical point for the rapid and efficient transfer of nano-based drugs from R&D to market is the translation of analytical methods from pre-clinical bench applications to clinical bed applications. For example, MRI, which is and will remain a first line diagnostic tool for many diseases like cancer and diseases of the brain, can be integrated with spectroscopy to build a multi-modal instrument that can be integrated into standard clinical practice. For example, MRI can be integrated with i) magnetic resonance spectroscopy (MRS) (Bolan et al., 2017) and/or special magnetic sensitive materials to combine imaging with image-guided drug delivery to assess tumor recurrence and treatment response (Kluza et al., 2012; Langereis et al., 2013), ii) NIR II (generally between 1,000 and 1,700 nm) or photoluminescence imaging to improve spatial resolution (Liu et al., 2014; Shen et al., 2016), iii) Raman and surface enhanced Raman spectroscopy (SERS) to replace *ex-vivo* histopathology and real-time guidance during surgery “intraoperatively” to accurately resect a tumor (Jermyn et al., 2015; Brusatori et al., 2017; Desroches et al., 2018; Zhang et al., 2019), iv) SERS for imaging of nanoparticles and real-time monitoring of drug release from the nanoparticles (Kircher et al., 2012; Ock et al., 2012; Zavaleta et al., 2013; Srinivasan et al., 2016; Tian et al., 2016; Zhang et al., 2019), and v) photo-acoustic imaging to improve spatial resolution and 3D imaging (Kircher et al., 2012). Fluorescence and SERS integration to achieve dual-mode imaging of nanoparticles at the disease site and to simultaneously quantitate drug release can be another fast and safe alternative to traditional destructive and label-based methods (Kang et al., 2013; Yang et al., 2014), however, it will be technically challenging as optical imaging has limited depth of penetration due to high tissue absorption and scattering and auto-fluorescence which contributes to background signal (De la Zerda et al., 2010). Gambhir et al. at Stanford University integrated MRI, photoacoustic, and SERS technologies (three-in-one) to develop a triple-modality imaging technology for treating brain cancer (Kircher et al., 2012). Unlike traditional gadolinium-based MRI, and fluorophore-based fluorescence imaging that requires multiple stereotactic injections of the contrast agents to guide neurosurgeons during pre- and intra-operative procedures, this triple modality technology will use a single injection of gadolinium-doped gold nanoparticles with a Raman tag to simultaneously achieve pre- and intraoperative 3-D whole brain imaging (MRI and photo-acoustic), and ultrasensitive and specific detection of tumor margins to allow accurate resection of the tumor (by SERS), by allowing the neurosurgeon to make real-time decisions by avoiding the need for waiting for histopathology. In contrast to the traditional approach of recruiting several hundred to thousands of patients in a clinical trial and then doing just a handful of measurements to collect a limited number of data/factors (Schork, 2015), the multi-modal technologies can enable us to collect high-quality and high-content information with minimum pain to the animal or human. To translate such multi-modality multi-factorial approaches, effective and efficient data analytics, and deep machine learning are important pre-requisites. The high content information from large datasets will allow doctors to make accurate diagnostic decisions (Tagare et al., 1997). Further, this multi-modal high-content technology could be leveraged to help us better understand and assess any nano-based drug under study in a clinical trial.

### Specificity and Efficacy

**Figure 1:**
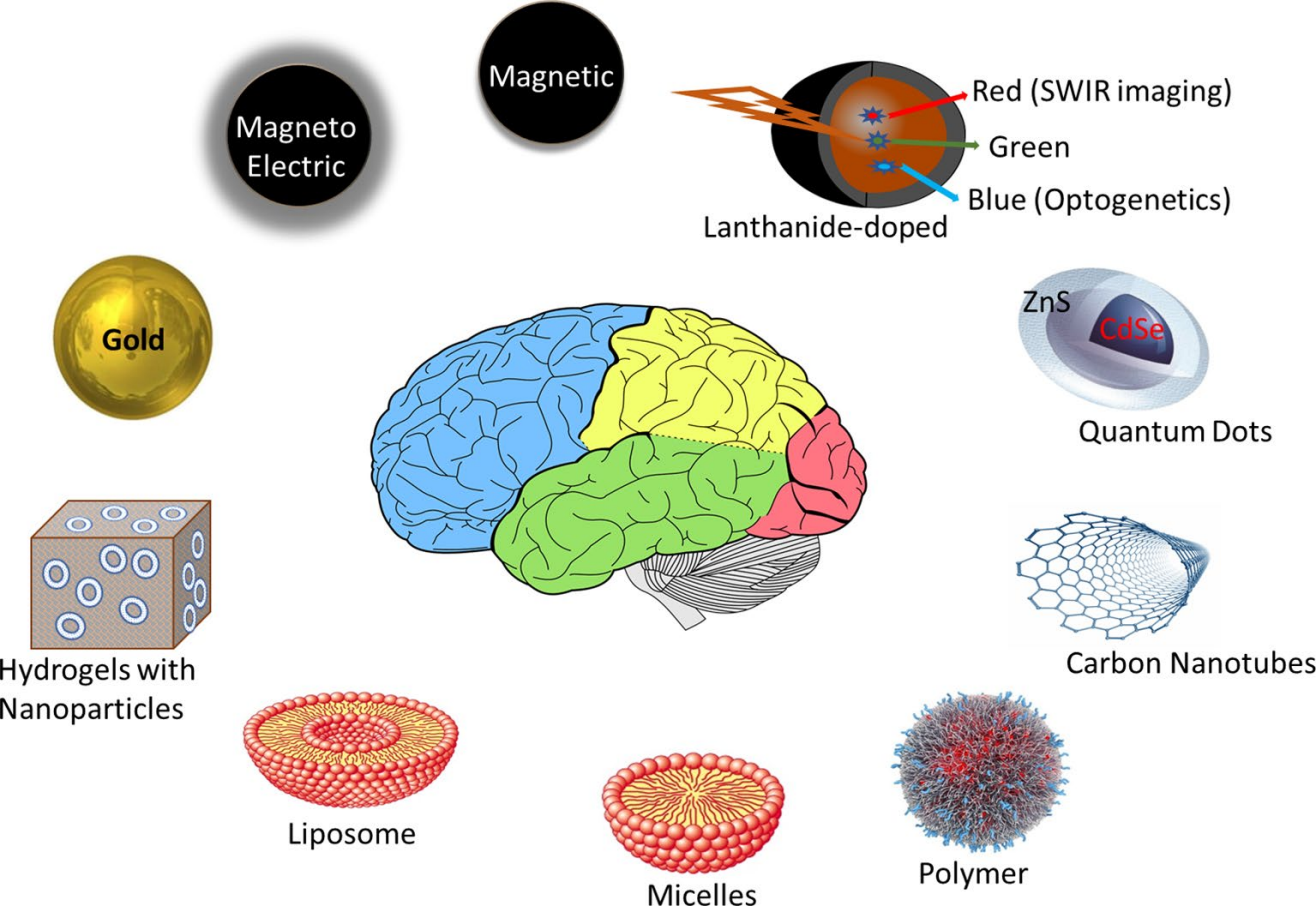
Examples of some of the most promising nano-materials used in clinical nano-pharmacology and/or image-guided nano-pharmacology research and development. Liposomes, micelles, and polymers are the most common nanomaterials in commercial nano-based drugs (>80% of the products). Metals nanoparticles like magnetic, magneto-electric, lanthanides, quantum-dots, and gold are most promising agents for multi-functional properties to achieve image-guided drug delivery.

**Figure 1** highlights nano-materials that are most commonly used in nano-pharmacology research and development. To have high specificity and efficacy, a nano-formulation should be able to i) encapsulate the required therapeutic dose of drug (require lower therapeutic dose than counterpart free drug treatment), and ii) deliver and release this drug specifically to the disease site (to achieve higher efficacy than free drug). These properties are achieved by specifically targeting the nano-formulation to the disease site. The approaches to control targeting and release properties can be broadly categorized as: passive (diffusion-based) and active (including facilitated). All the existing commercial nano-based drug in the market (excluding antibody-drug conjugates which are not generally considered “particles”) are based on passive targeting (Rosenblum et al., 2018). The passive targeting approach is based on the inherent physico-chemical properties of the nano-formulation and the biological environment of the disease site. Usually, the size of the nano-formulation for passive diffusion should be less than or equal to 100 nm for cancer (EPR effect), and much smaller than 100 nm for brain diseases (for transmigration across tight junctions between the endothelial cells of the blood-brain-barrier). Although larger nanoparticles can be delivered across the blood-brain-barrier using active delivery approaches, passive diffusion is most efficient for nanoparticles 20 nm or smaller because endothelial clefts in tight junctions have transient opening restricted to 20 nm width (Masserini, 2013). The particles should have long circulation time to avoid their uptake by the Reticulo-Endothelial System (RES), which is the body’s natural clearance pathway for any foreign material. As summarized by Professor Yechezkel “Chezy” Barenholz, the key inventor of the Doxil^®^ technology, these physico-chemical properties of the nano-formulations were important drivers during his 20 year journey in bringing this first FDA-approved nano-based drug to the market in 1995 (Barenholz, 2012). In order to overcome the limitations of his original design that failed clinical trials in 1987 he made the following changes to tune the physico-chemical properties of the Doxil^®^: i) changed the oligolamellar liposome chemistry to unilamellar liposomes (to bring the size to less than 100 nm), ii) developed a stealth nano-formulation by adding a polyethylene glycol coating around the liposome to increase circulation time, avoid opsonization, and avoid the RES, and iii) incorporated an active gradient approach for achieving a high payload of the drug inside the liposomes.

Indeed, the physico-chemical properties of the nano-formulations are critical for targeting and release (efficacy). However, the targeting property of the nano-formulation can be improved to deliver drug more efficiently by modifying the physico-chemical properties of the nano-formulation to more specifically target disease cells. For example, the classical approach is conjugating a ligand, such as antibodies, aptamers, or small molecules to the nanoparticles that binds specifically to the biomarkers that are abundant on the diseased cells. Technically speaking, ligand-mediated facilitated diffusion is expected to deliver high specificity over passive diffusion; however, it is practically not always the case. For example antibody targeted Doxil^®^ failed to perform *in vivo*, in fact, its efficacy was even inferior to passively targeted Doxil^®^ (Jakoby et al., 2015). An interesting least-component based approach of targeted drug delivery using bioactive polymers, wherein polymers are engineered to have targeting moieties, replacing the need of conjugating additional targeting ligands like antibodies, was developed by Prof Kathryn Uhrich (Gu et al., 2014; Zhang et al., 2015). Her team formulated a library of sugar-based amphiphiles with geometries similar to liposomes, hydrophobic core (to encapsulate drug), and hydrophilic shell, wherein the shell is engineered to have targeting moieties (Gu et al., 2014) and then screened these bioactive polymers for their anti-cancer and anti-Parkinson activities (Bennett et al., 2016; Gu et al., 2017).

There is a growing interest in metal nanoparticles and the triggered release approach that is based on release of drug in response to a stimulus like heat, light, enzyme, pH, etc. (Sharma et al., 2015; Srinivasan et al., 2016; Hua et al., 2018). FDA-approved ThermoDox^®^ (Gray et al., 2019) and European-approved NanoTherm^®^ (Maier-Hauff et al., 2011) are two successful examples of nano-based drugs based on thermo-therapy, which is, using heat to ablate a tumor (Nanotherm^®^) or trigger drug release at the target site (ThermoDox^®^). Both products use a thermo-sensitive nanomaterial to produce heat in response to focused ultrasound (ThermoDox^®^) or an alternating magnetic field (NanoTherm^®^). NanoTherm^®^ from MagForce (Germany) is particularly interesting because it is the first nanopharma product to treat glioblastoma, uses iron oxide nanoparticles, is approved in Europe, and is now under clinical trial in the USA. However, NanoTherm^®^ technology is invasive because it requires a stereotactic injection of iron-oxide based magnetic nanoparticles directly into the brain. While metal nanoparticles may be an excellent choice to simultaneously achieve cancer diagnosis and therapy, i.e., theranostics (Sharma et al., 2015), to our knowledge, currently there is not a single FDA-approved metallic nanoparticle-based drug for treating cancer or brain diseases (**Table 1**).

Based on a somewhat similar concept to ThermoDox^®^ and NanoTherm^®^, except being based on a heating mechanism to achieve benefits, our group has developed a non-heating active delivery approach by using novel magneto-electric nanoparticles (MENs) to achieve non-invasive magnetically-guided targeted therapy for the brain (Yue et al., 2012; Kaushik et al., 2016), cancer (Guduru et al., 2013; Rodzinski et al., 2016), and HIV (Nair et al., 2013). Our MENs strategy does not use any targeting and responsive ligands, rather, it is based on the physical phenomenon of magnetic delivery and low frequency non-heating magneto-electric-actuation to release the drug (Stimphil et al., 2017; Golovin et al., 2017). Compared to passive and ligand-mediated diffusion, our physical targeting approach (a true active delivery) is: rapid, just a few hours to deliver nanoparticles to the brain, and on-demand triggered drug release from these particles by controlling frequency of the electromagnetic field generated by the MENs. In addition to an iron oxide magnetic component (as in ThermoDox^®^ and NanoTherm^®^), our MENs also have a component that allows magneto-electric-actuation to release the drug without any heating side-effect (Stimphil et al., 2017). Currently, we are integrating our MENs strategy with MRI to do image-guided drug delivery to treat neuroHIV in non-human primates. Another interesting example of a non-heating non-invasive physical approach approved by the FDA and Europe to treat cancers, including glioblastoma, is tumor treating electric fields (TTFields) (Stupp et al., 2012). The TTFields acts by triggering mitotic cell death of cancer cells. Compared to NanoTherm^®^ technology for glioblastoma that requires invasive delivery of nanoparticles inside the brain and a large instrument (nanoActivator^®^), Novocure’s TTFields requires just a simple portable Optune^®^ system and no nanoparticles. The Optune^®^ system weighs 2.7 pounds and consists of a small electric field generator and the transducer arrays.

### Affordability

Development and translation of nano-pharmacology technology to a product in the market is extremely difficult, extremely long, and extremely expensive. Roughly, it takes $750 million and 8 years to bring a new cancer drug from R&D to the market (Prasad and Mailankody, 2017). Less than 50% of the drugs in Clinical phase III trials are approved by the FDA (Hwang et al., 2016), therefore, investment in nano-pharmacology is high risk, but high reward. Although the risk (cost) associated with every drug development is high, the risk and cost is particularly high in the development of a nano-based drug because, i) subject matter experts are scarce, ii) scale-up of the nano-formulation process at a manufacturing level is technically challenging, and iii) the regulatory pathway is vague. No doubt, scientists have sufficient support from non-profit and for-profit organizations to test and demonstrate their unique proof-of-concept nano-pharma technologies. However, these original innovators (typically the scientists and engineers in universities) require strong industrial and fiscal support to translate their nano-pharma technology to market.

Over the history of nano-pharmacology research and commercialization that started with development of the first commercial product, Doxil^®^ in 1979 (in which the first clinical trial was a failure), the scientific community has not learned as much as they should have about the technical and fiscal obstacles in nano-pharmacology (Barenholz, 2012; Barenholz, 2012). A team of professors from the USA, Israel, and Canada invented the proof-of-concept Doxil^®^, which was later developed by a few startup companies and eventually bought by a global pharma, company Janssen Pharmaceutical (part of J&J), before reaching the market (Barenholz, 2012). J&J was the exclusive supplier of Doxil^®^ (Caelyx^®^ in Europe) before the patent expired in 2010. Soon after the expiry of the patent, Janssen shut the manufacturing lab and there was a Doxil^®^ shortage. Shortage of Doxil^®^ is still a major concern. We can relate this shortage issue with the expiry of the patent and the amount of money and time invested in R&D and regulatory approval to revenue when trying to manufacture Doxil^®^ at low cost (generic Doxil^®^). Since the first shortage of Doxil^®^ in 2011, there have been a few examples of generic Doxil^®^ in the market, Sun Pharmaceutical in India being the lead manufacturer of Lipodox^®^ (generic Doxil^®^) for the USA and Europe (Barenholz, 2012; Hale and Goldberg, 2012; Bhowmik et al., 2018). Generic Copaxone^®^ to treat Multiple sclerosis is another of the latest successful examples of making nano-pharma drugs affordable.

The experience of Accurins^®^, a class of novel targeted therapeutics developed by BIND Therapeutics, represents another telling example to support our point on the fiscal challenges associated with nano-pharma (Van der Meel et al., 2017). The BIND technology, based on Accurin polymer nanoparticles to improve delivery of docetaxel (BIND-014) (Autio et al., 2017) and Aurora B Kinase inhibitor (AZD2811) (Ashton et al., 2016), was originally developed by a collaboration between MIT and Harvard and was able to win a wider collaboration of world class industrial partners like AstraZeneca, Amgen, Merck, etc. However, due to difficulty in phase II clinical trials of BIND-014 (ClinicalTrials.gov identifier or trial# NCT02479178) and AZD2811 (trial #NCT03366675), and the associated fiscal loss and confidence of the industrial partners, BIND eventually declared bankruptcy (Van der Meel et al., 2017; Nanalyze, 2017). In a recent bid, Pfizer bought all of the BIND assets for ∼$40 million in an effort to translate the still very promising BIND nano-pharmacology technology to market (Genetic Engineering and Biotechnology News, July 27, 2016). The BIND technology is once again recruiting subjects for phase I clinical trials of AZD2811 (trial#NCT02579226). The eventual success of this technology will be very welcome to patients, the industry, and the field in general. This lesson shows that the failure of any new drug could be due to overly ambitious, or poorly defined clinical endpoints, or due to investors losing confidence. This can happen even if a drug is technically efficacious.

Personalized or precision nano-based drugs, is another hot topic of research that makes nanomedicine more exciting and luring to researchers and investors. For example, there is only <10% chance (1 in 16) that a patient will respond to Copaxone^®^, the nano-based drug for multiple sclerosis (Schork, 2015). No doubt, precision medicine has much higher chances of successful treatment than classical “imprecision” medicine (Schork, 2015). Moderna Inc. USA which recently received fast track FDA approval for a zika vaccine and has partnered with Merck pharma to develop a personalized cancer vaccine for high risk melanoma patients, clinical phase II in progress (trial#NCT03897881).

The battle between the University of California, Berkeley and the Broad Institute at MIT over CRISPR technology is a “learning experience” for everyone (Nature news, 10 Sept 2018). CRISPR-Cas9 gene editing technology was first demonstrated in bacteria by scientists at UC Berkeley. However, after a few months scientists at Broad Institute significantly improved the technology by publishing their research on the potential of this technology in editing plants and animal cells, including humans to improve their health. We are in full agreement with the court’s decision on not giving exclusive right of CRISPER-Cas9 technology to UC Berkeley, who spent millions of dollars in litigation against Broad Institute. In our opinion, although the core of the technology might be the same, the improvement to translation for a plethora of real-world applications was a significant achievement by Broad Institute. The point is that science and innovation should not stop even when a technology is protected. In fact this court decision has motivated many scientists to investigate alternative strategies to Cas9 for CRISPER gene editing technology. Also, we have seen a growing number of grant proposals and research papers on the personalization of nanomedicine. We wonder, when nano-based drugs are already expensive and require sophisticated technology (several parameters to control simultaneously) and its hard to reproduce, how feasible it is going to be to personalize these products (custom formulation for every patient)? In our view, no doubt, personalization of nanomedicine is technically feasible and very important; however, our concern is the cost factor. But, some developments are encouraging. For example, Foundation Medicine, Inc. is the first FDA-approved American Public health company that offers genomic testing to patients at ∼$5,000 to $7,500, making precision medicine for cancer closer to becoming a reality https://www.foundationmedicine.com/.

### Vague Regulatory Landscape

In addition to the requirement for technical expertise and high cost associated with manufacturing of nano-based drugs, a major hurdle in their commercialization is the difficult regulatory landscape (Bawa, 2011; Etheridge et al., 2013; Gaspani and Milani, 2013; Bawa et al., 2016). FDA’s regulatory landscape for nano-based drugs is vague and the approval process is very long, which could lead to delay or loss of commercial nano-based drugs (Bawa, 2011; Gaspani and Milani, 2013; Bawa et al., 2016). For example, there is no regulatory definition of a nano-based drug, as a matter of fact, for any nano-based product (Food and Drug Administration, 2014). There is no consensus for a standard method to measure bioequivalence (therapeutic efficacy) of a new drug with respect to a reference drug in the market (Davit et al., 2009; European Medicines Agency, 2013). Compared to FDA regulations in the USA that rely on a classical approach of comparing a very limited number of pharmacokinetics parameters, usually area under the curve (AUC) or maximum peak concentration (Cmax) of the drug in plasma (Davit et al., 2009), regulations in the European Medicine Agency consider several pharmacokinetic parameters to get detailed information on bioequivalence (European Medicines Agency, 2013). There is a financial, and even a scientific, urge towards simplifying regulations; however, it is understood that even a small change in physico-chemical properties of nanoparticles carrying a drug can lead to major safety and efficacy issues (Mamidi et al., 2010).

Although the FDA established the Nanotechnology Task Force in 2005 to streamline the regulatory pathway for nano-products, the FDA is going to continue with their precautionary approach. As scientists, we agree with the FDA’s precautionary approach in approving nano-based drugs because: 1) they are more complex than traditional drugs, 2) nanotechnology has some negative public opinion, and 3) there is insufficient, and sometimes, contradictory information on safety and efficacy findings on nanotechnology products (Editorial, 2007). Nevertheless, a consensus is needed as to the precise definition of a nano-based drug, and what, if any, unique characteristics will be required for consideration of approval for use in humans. The FDA’s Nanotechnology Task Force solicited public opinion to gather scientific comments on regulatory policy issues that concerns the FDA, and proposed recommendations to improve the nanotechnology regulatory landscape (Nanotechnology Task Force and US Food and Drug Administration, 2007). Undoubtedly, the current regulatory landscape for nanotechnology products is not adequate, and continuous improvements are needed to revise guidance documents on nanotechnology products including nano-pharmacology and biologics (US Food and Drug Administration, 2014; US Food and Drug Administration, 2017).

## Discussion

Nanotechnology is exciting and promising. Although slow to move forward, there have been successes in the commercialization of nano-based drugs, evident by the availability of ∼20 nano-based drugs including a few generic nano-based drugs and personalized medicine. There are even more antibody and polymer drug conjugates available. However, as innovators, investors, healthcare providers, and consumers we all need to understand that nano-based drug development and commercialization is an extremely difficult, long, and expensive process. To safely and efficiently translate the benefits of nano-pharmacology in alleviating global health problems like cancer and brain diseases, global efforts and public-private partnerships are critical so that we can share the scarce resources, the associated risks, and the responsibilities to develop a global standard regulatory framework. Although there are several technical and non-technical challenges in the critical path to making nano-based drugs available and affordable to all, in agreement with some other experts in this field, these two points are most important in our opinion. First, there is no label-free quantitative analytical technology available in clinics to, in real-time, monitor the bio-distribution of nanoparticles and the drug release (indicators of efficacy) (Sanhai et al., 2008). The real-time (pain-less) and accurate (label-free) information on bio-distribution are important indicators of product efficacy. Second, undoubtedly, pre-clinical animal studies are a pre-requisite to clinical testing and a good sample size is critical to investigate before coming to a conclusion on safety and efficacy. However, measuring just a handful of factors, even in a large sample size, is not sufficient (Schork, 2015). We should consider developing predictive models and pain-free analytical methods to gather high content information from each animal or human under trial. We should learn from the journey of the first nano-based drug Doxil^®^; from failed clinical trial to continuous improvement, and from the Doxil^®^ shortage to its generic counterpart Lipodox^®^ (Barenholz, 2012). In addition to development and protection of proprietary nano-based drugs, we should also focus on the timely development of their generic versions as the patent of all the proprietary nano-based drugs will eventually expire (Davenport, 2014). Finally, while investors need a financial return to encourage further research and development, ultimately, nano-based drugs need to be available and affordable to all.

## Data Availability Statement

Publicly available datasets were analyzed in this study. This data can be found here: https://clinicaltrials.gov/.

## Author Contributions

VB, AK, and AM conceived the idea/topic for the opinion. VB and AM wrote the manuscript. AK, ZK, and MN reviewed the manuscript and contributed to the intellectual scientific content of the manuscript domain specific expertise.

## Conflict of Interest

The authors declare that the research was conducted in the absence of any commercial or financial relationships that could be construed as a potential conflict of interest.
